# Pelvic and Digital Rectal Examinations to Evaluate Lower Urinary Tract Symptoms

**DOI:** 10.1001/jamanetworkopen.2026.9267

**Published:** 2026-04-27

**Authors:** Kathryn McLeod, Marina Youssef, Margaret Rogers, Richard Grills

**Affiliations:** 1 Department of Urological Surgery, Barwon Health, University Hospital, Geelong, Australia; 2 Department of Surgery, School of Medicine, Deakin University, Geelong, Australia; 3 Department of Surgery (Austin), University of Melbourne, Heidelberg, Australia; 4 Surgical and Critical Care Directorate, Barwon Health, University Hospital, Geelong, Australia

## Abstract

**Importance:**

A thorough examination of the patient is a crucial component of providing high-quality care and is regarded as best practice in the assessment of female patients presenting with lower urinary tract symptoms (LUTS). Nevertheless, anecdotal reports suggest that pelvic examinations are infrequently performed in the outpatient setting, raising important questions regarding adherence to established standards of care.

**Objective:**

To explore the practices and attitudes of urologists toward pelvic examination for patients with LUTS.

**Design, Setting, and Participants:**

In this qualitative, mixed-methods study, a structured 9-question survey about digital rectal and pelvic examination practices was distributed between November 19, 2023, and May 19, 2024, to urologists and urology trainees via the Urological Society of Australia and New Zealand newsletter across Australia and New Zealand. The end of the survey invited clinicians to participate in a semistructured interview to further discuss their responses.

**Main Outcomes and Measures:**

The primary outcomes were urologist-reported responses to the survey assessing the numbers and percentages of male and female clinicians who routinely performed pelvic and digital rectal examinations for patients with LUTS at their initial appointment and qualitative semistructured interview responses to identify themes associated with clinician attitudes toward pelvic examinations.

**Results:**

Of 553 consultant urologists and 100 urology trainees sent the survey, 74 participants responded, a response rate of 11.8%. A total of 74 clinicians (46 [62.2%] male), comprising 63 urologists and 11 urology trainees, completed the survey. While 89.1% (95% CI, 80.1%-98.1%) of male clinicians and 92.9% (95% CI, 83.3%-100.0%) of female clinicians consistently performed digital rectal examinations for male patients presenting with LUTS, only 8.7% (95% CI, 5.5%-16.8%) of male clinicians routinely conducted pelvic examinations for female patients compared with 85.7% (95% CI, 72.8%-92.9%) of female clinicians (*P* < .001). In total, 10 semistructured interviews were conducted. Two major themes were identified: fear, including medicolegal reprimand, clinician and patient discomfort or reluctance, and failure to recognize pathology; and barriers to pelvic examinations, including perceived poor utility, limited access to chaperones, and concerns for patient discomfort.

**Conclusions and Relevance:**

Findings of this study suggested that male clinicians were less likely than female clinicians to perform pelvic examination for female patients who presented with LUTS. Barriers to pelvic examination need to be addressed to enable female patients to receive optimal care. Focused training may help prevent unnecessary operative procedures and improve patient outcomes.

## Key Points

**Question:**

What are the practices and attitudes of urologists toward pelvic examination for patients with lower urinary tract symptoms?

**Findings:**

This qualitative, mixed-methods study surveyed 74 urologists and trainees and found that male clinicians were significantly less likely than female clinicians to perform pelvic examinations for female patients presenting with lower urinary tract symptoms.

**Meaning:**

These findings suggest that female patients are less likely to receive a comprehensive examination when seeing a male clinician and that barriers to pelvic examination need to be addressed for female patients to receive optimal care.

## Teaser

This qualitative, mixed-methods study investigates the practices and attitudes of female compared with male urologists toward pelvic examination for patients with lower urinary tract symptoms.

## Introduction

Lower urinary tract symptoms (LUTS) encompass storage, voiding, and postmicturition symptoms, including frequency, urgency, nocturia, urinary incontinence, hesitancy, intermittency, slow stream, straining, urinary spraying, and terminal dribble.^1^ Pelvic examinations play a critical role in the evaluation of patients presenting with LUTS because a range of anatomical causes are identifiable on examination, including prostate abnormalities, pelvic organ prolapse, urogenital atrophy, pelvic floor muscle function, stress incontinence, complications of or prior pelvic surgery, and pelvic masses. Identification of these underlying causes may not be apparent from history alone yet may substantially influence management decisions.^1,2^ Accordingly, international continence and urological guidelines recommend routine pelvic examination as a core component of the initial assessment of patients with LUTS.^3,4,5^

However, there is anecdotal evidence that many urologists and urological trainees do not routinely perform pelvic examinations on female patients presenting with LUTS. This is supported by a US study conducted in a urology clinic that demonstrated that male patients were 9 times more likely than female patients to undergo a genital exam in the evaluation of lower urinary tract symptoms.^6^ Despite a lack of further evidence from a urological perspective, this sex disparity in performing pelvic examination for female patients is also reported in studies involving emergency department (ED) physicians, who had concerns regarding the clinical utility of the examinations in the ED, lack of confidence and training, and inadequacy of the setting, including privacy, equipment, and time pressures.^7^ ED resident perspectives revealed that male residents reported barriers, including a lack of training, general dislike, and concern that patients would prefer female clinicians.^8^

Given the important role of pelvic examination in the clinical assessment and evaluation of LUTS and the limited data on clinician perceptions from a urological perspective, this study examined the practices of urologists and urology trainees in Australia and New Zealand and their attitudes toward pelvic and digital rectal examination (DRE) in the evaluation of LUTS.

## Methods

We performed a qualitative, sequential, mixed-methods study designed to investigate the practices and attitudes of urologists and urology trainees toward performing pelvic examinations. The study consisted of an initial structured survey followed by semistructured interviews. This study followed the Standards for Reporting Qualitative Research (SRQR) reporting guideline^9^ and was approved by the Barwon Health Human Research Ethics Committee. All participants in the survey and interviews provided written informed consent.

Eligible participants included urologists and accredited surgical and education training urology trainees. There are 553 consultant urologists and 100 urology trainees across Australia and New Zealand,^10^ 86% identified as male and 14% identified as female. A sample size calculation was conducted based on the hypothesis that 90% of female urologists and 50% of male urologists perform pelvic examinations. Using a 2-proportion *z* test, a significance level (α) of .05, and power (1 – β) of 0.80, we determined that a minimum of 17 survey participants in each sex group was required to detect this 40-percentage point difference.

A structured 9-question survey (eAppendix 1 in Supplement 1) was distributed to all eligible participants via the Urological Society of Australia and New Zealand (USANZ) newsletter over a 4-week period. The survey was opt-in, anonymous, and administered through the REDCap platform. At the conclusion of the survey, participants were invited to participate in a semistructured interview. Among those who consented to interview, we used purposive sampling to select a variety of participants, ensuring diversity in terms of training background, sex, and attitudes expressed in survey responses.

Semistructured interviews were conducted by one of us (M.Y.) via Zoom, telephone, or in person using an interview guide (eAppendix 2 in Supplement 1). Audio recordings were transcribed and deidentified by M.Y., and data were deidentified prior to reflexive thematic analysis.^11^ M.Y. inductively created preliminary codes. Independently, another member of our team, K.M., inductively analyzed and coded 4 transcripts. K.M. cross-checked these 4 sets of codes for alignment. K.M. generated initial themes, and then all researchers met to further refine themes based on the data. After 10 interviews, we considered that data sufficiency^12^ was reached.

Authors brought different perspectives to the research. M.Y. is a female unaccredited urology registrar. K.M. is a female urologist subspecializing in female and functional urology as well as qualitative research. R.G. is a male urologist specializing in robotic oncology.

### Statistical Analysis

The survey collected both demographic and binomial data, including practices related to pelvic and DREs. Descriptive statistics were used to summarize participant responses. Group comparisons between female and male respondents were conducted using χ^2^ tests or Fisher exact tests for categorical data, where appropriate. A 2-sided *P* < .05 was considered statistically significant. StataBE, version 18 (StataCorp LLC) was used for these analyses. NVivo, release 1.6.2 (QSR International Pty Ltd) was used for interview data management.

## Results

Between November 19, 2023, and May 19, 2024, of 553 consultant urologists and 100 urology trainees sent the survey, 77 participants responded. There were 3 respondents noted to have placed 2 separate entries. Their secondary answers were discounted, leaving 74 responses (28 [37.8%] female and 46 [62.2%] male), a response rate of 11.8%. There were 63 urologist (23 [36.5%] female, 40 [63.5%] male) and 11 urology trainee (5 [45.5%] female, 6 [54.5%] male) responses. Urologists were asked to list their primary subspeciality, with many opting for more than 1 subspeciality (Table 1). A total of 24 urologists reported functional urology; of them, 17 were female and 7 were male (*P* < .001).

**Table 1.** Subspecialties of Consultant Urologists Surveyed

Subspecialty	Participants, No. (%)		*P* value
	Male	Female	
No./total No.	40/63	23/63	NA
Functional	7 (18)	17 (74)	<.001
Robotic surgery	14 (35)	6 (26)	.46
Oncology	23 (58)	5 (22)	<.006
General urology	13 (32)	2 (9)	.03
Stones and endourology	7 (18)	5 (22)	.68
Reconstructive urology	7 (18)	4 (17)	.99
Andrology	6 (15)	2 (9)	.47

Abbreviation: NA, not applicable.

**Figure 1.**
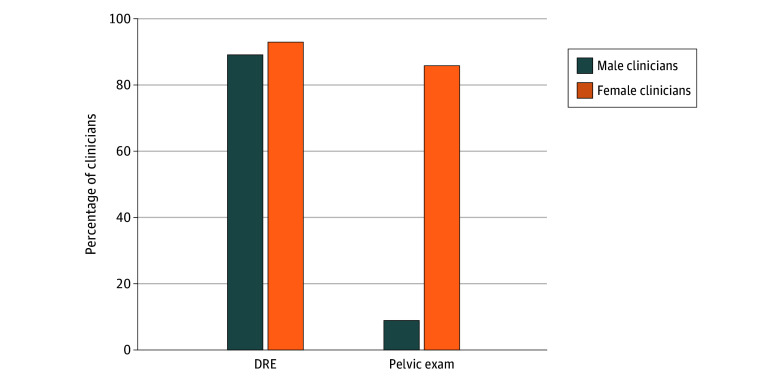
Bar Graph Showing the Percentages of Male and Female Clinicians Who Perform Digital Rectal Examinations (DREs) and Pelvic Examinations More Often Than 75% of the Time for Patients With Lower Urinary Tract Symptoms at the Initial Appointment

The majority of participants, 89.1% (95% CI, 80.1%-98.1%) male and 92.9% (95% CI, 83.3%-100.0%) female, reported performing a DRE more often than 75% of the time for male patients who presented with LUTS on initial presentation (*P* = .60). Conversely, only 8.7% (95% CI, 5.5%-16.8%) of male clinicians reported performing a pelvic examination more often than 75% of the time for female patients who presented with LUTS compared with 85.7% (95% CI, 72.8%-92.9%) of female clinicians (*P* < .001) (Figure 1). Reasons provided as barriers for DREs and pelvic examinations (Table 2) included the need for chaperones, no clinical indication, deferral until procedure, patient reluctance, and time constraints.

**Table 2.** Reasons Provided by Male and Female Clinicians to Omit DRE and Pelvic Examinations

	Participants, No.	
	Male (n = 46)	Female (n = 28)
Reasons to omit DRE		
No. (%)	5 (10.9)	2 (7.1)
Virtual appointment	3	0
Poor sensitivity, prefer imaging	2	0
Not indicated for LUTS assessment	2	0
Patient declined	0	1
Not relevant to practice (pediatric)	0	1
Reasons to omit pelvic exam		
No. (%)	42 (91.3)	4 (14.3)
Defer to procedure (eg, cystoscopy, EUA, and urodynamics)	15	3
Need for chaperone	15	0
Clinically not indicated or would not change management	12	0
Perceived medicolegal risk or misconstrued by patient	5	0
Not their patient demographic	4	0
Select patients only	4	0
Patient reluctance	3	0
Time constraints	2	0
Clinician discomfort or awake patient	1	0
Lack of role modeling or training	1	0
Refer to physiotherapist to conduct	0	1

Abbreviations: DRE, digital rectal examination; EUA, examination under anesthesia; LUTS, lower urinary tract symptoms.

**Figure 2.**
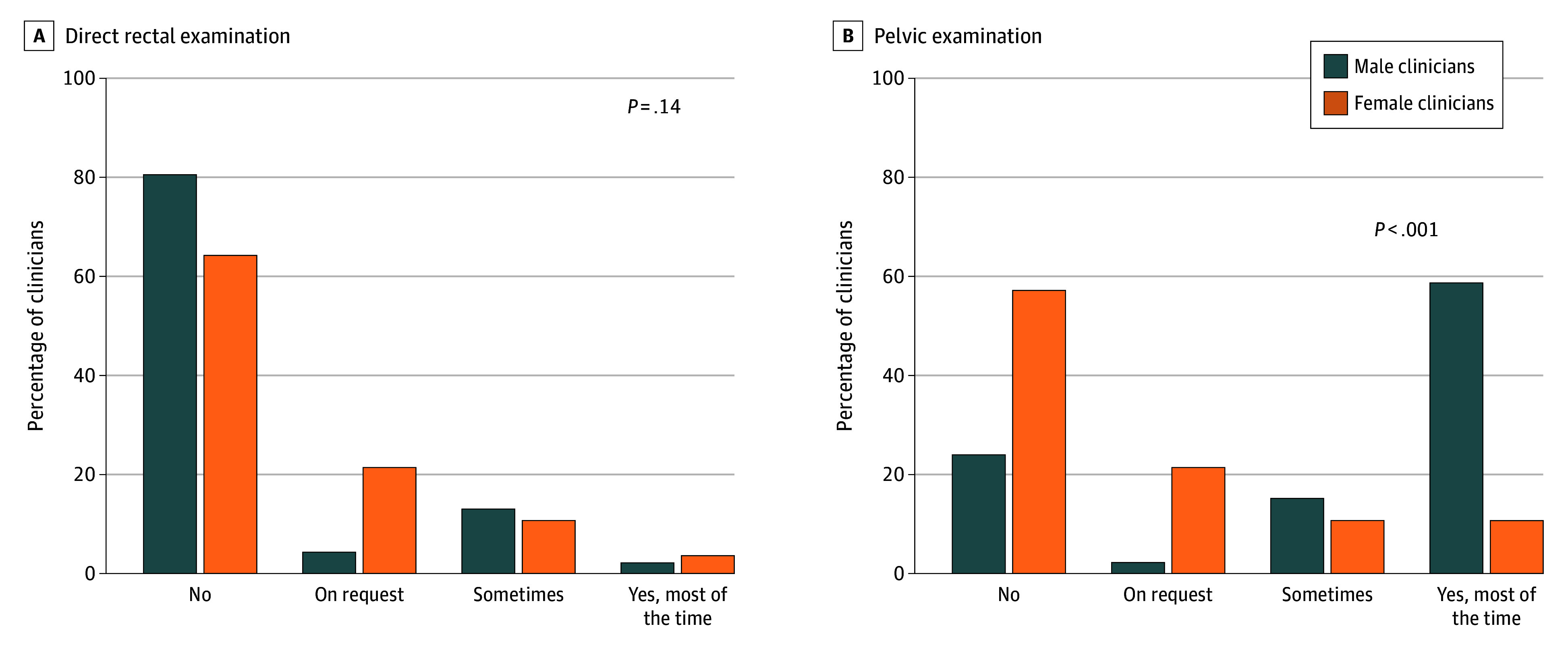
Bar Graphs Depicting the Percentages of Chaperone Use When Performing Digital Rectal Examinations and Pelvic Examinations Between Female and Male Clinicians

There was no significant difference in chaperone use between male and female clinicians conducting DREs (2.2% [95% CI, 0.0%-6.4%] vs 3.6% [95% CI, 0.0%-10.4%]; *P* = .14) (Figure 2). In contrast, male urologists were significantly more likely than female urologists to use chaperones during pelvic examination (58.7% [95% CI, 44.5%-72.9%] vs 10.7% [95% CI, 0.0%-22.2%]; *P* < .001). Among trainees, 16.7% (95% CI, 0.0%-46.5%) of males vs 0.0% (95% CI, 0.0%-43.4%) of females used chaperones routinely for DRE (*P* > .99), while 66.7% (95% CI, 28.9%-100.0%) of males vs 20.0% (95% CI, 0.0%-55.1%) of females routinely used chaperones for pelvic examinations (*P* = .24).

A total of 10 semistructured interviews took place, which included 7 males and 3 females, 8 urologists, and 2 trainees. Of these participants, 4 identified functional urology as their subspecialty. The mean (range) length of interviews was 33 (14-45) minutes. Based on the qualitative data collected in the interviews, 2 themes were identified, including barriers to performing pelvic examinations and fear. Themes and subthemes are outlined in the Box.

Box. **Themes and Subthemes on the Approaches of Urologists and Trainees to Pelvic Examinations**

**Barriers to performing pelvic examinations**

Logistically challenging
Perceived poor utility of pelvic examinations
Lack of training and role modeling from senior colleagues

**Fear**

Fear of medicolegal reprimand
Fear of clinician and patient discomfort or reluctance
Failure to recognize pathology

### Barriers to Performing Pelvic Examinations

This theme encompassed a range of challenges that limited performing pelvic examinations in outpatient settings. While some participants agreed that there were no excuses to omit a pelvic examination, many participants described the following barriers to performing examinations: logistical challenges, perceived poor utility of pelvic examinations, and a lack of structured training and role-modeling from their senior counterparts.

#### Logistical Challenges

Participants frequently cited logistical factors, including lack of chaperones, time constraints, and inadequate clinic infrastructure as barriers to performing pelvic examinations. Some reported limited access to chaperones in private consultation rooms as a substantial obstacle. Many clinicians thought it more convenient and logistically easier to perform a pelvic examination at the time of a flexible cystoscopy. “[I] almost never [perform a pelvic examination in my rooms]. I tend to book them for a flexible [cystoscopy], which is almost always indicated anyway.” (Participant identification [ID] 2, male urologist; robotic surgery and oncology subspecialties). “In my private rooms where I don’t have access to a chaperone, it’s definitely a minority [of females that get a pelvic exam].” (Participant ID 3, male urologist; robotic surgery and oncology subspecialties).

The additional time required to perform a pelvic examination, particularly in older female patients with limited mobility was also mentioned as a challenge. “I’m willing to compromise on the quality of the examination for a timely examination.” (Participant ID 10, male trainee).

Many participants indicated that these practical limitations often served as excuses or justification for other clinicians avoiding the examination, which would not meet the standard of best practice for patient care. “We were always told, ‘If you don’t put your finger in it, you put your foot into it.’ These are all sort of barriers that have been completely overcome by the average gynecologist.” (Participant ID 1, female urologist; functional and reconstructive urology subspecialities). “I think a lot of people use [needing a chaperone] as an excuse, rather than…actually the patient being particularly worried about it. And again, you’re just not offering the patient the…best diagnosis.” (Participant ID 4, female urologist; functional, stones, and general urology subspecialties).

#### Perceived Utility of Pelvic Examinations

Many male participants perceived that omitting pelvic examinations was unlikely to alter clinical management. Many believed the risk of missing a malignant lesion was minimal and that LUTS were usually indicative of benign pathology. They perceived pelvic examinations for the evaluation of LUTS as low yield, potentially uncomfortable for patients, and carrying a risk of legal repercussions due to a perception of patient violation. In contrast, they generally considered DREs essential for all male patients as a screening tool for prostate cancer, regardless of their presenting symptoms, and did not consider patient discomfort as a deterring factor to doing so. “The one good thing…is that no one ever died from being wet.” (Participant ID 8, male urologist; general, functional, and reconstructive urology subspecialties).

However, this perception was not expressed by all. Many participants expressed that pelvic examinations were a vital part of a complete and thorough examination and were needed to detect red flags. “All too often people use these sorts of things as excuses to make shortcuts. And as I say, 98% of the time, you’ll get away with it. And then 2% of the time, you’ll look like an idiot. It’s just not worth it.” (Participant ID 7, male urologist; general).

#### Training and Role Modeling From Senior Colleagues

Some participants stated that they do not perform pelvic examinations because it was never role-modeled by their supervisors during their training years as a routine assessment for LUTS. Further to this, some believed that there was little teaching and low emphasis placed on the importance of pelvic examination during their training. Study participants who were current trainees echoed this sentiment. “I think for the first three-quarters of my training, it was completely accepted that you didn’t have to examine a woman who came in with hematuria or urinary symptoms. I never had a consultant ask me what [I] saw on examination.” (Participant ID 6, male trainee).

In contrast, participants who did routinely perform pelvic examinations often attributed their confidence to having trained with functional urologists. These experiences allowed for practical, hands-on learning that helped normalize the examination. “At this point of my training onwards, I feel very comfortable and confident doing pelvic examinations. So for learning, it’s working with…functional urologists.” (Participant ID 10, male trainee).

### Fear

Fear emerged as a prominent underlying factor contributing to the avoidance of female pelvic examinations. This fear was rarely rooted in direct experience but was more commonly described as a learned behavior, modeled by senior colleagues and perpetuated through training environments associated with a culture of caution and avoidance of pelvic examinations. “And I think it’s, well, unfortunately we’ve been told, or we’ve learned from our colleagues, that you’ve got to be careful…doing a [vaginal examination] on a female.” (Participant ID 8, male urologist; general, functional, and reconstructive urology subspecialties).

#### Medicolegal Consequences

Fear of medicolegal repercussions emerged as an important deterrent to performing pelvic examinations. One participant reported a direct negative experience when they were a trainee, which did not escalate. Notably, the practice of obtaining informed consent for a pelvic examination was rarely mentioned as a strategy to mitigate these concerns and was not considered sufficiently protective. Instead, the fear of the purpose of the examination being misunderstood remained a dominant concern for clinicians who did not routinely perform pelvic examinations. “As a male, there’s no way I would remotely consider doing a vaginal examination without a chaperone.” (Participant ID 5, male urologist; andrology, functional, and reconstructive urology subspecialties).

Contrastingly, this was not voiced by any female study participant when discussing male genital examination. Many acknowledged the sensitive nature of these examinations and emphasized the need to approach them with care. “Why do we differ between female patients and male patients? We’re willing to stick our fingers up the bum of male patients but we’re not willing to do a proper vaginal examination on female patients.” (Participant ID 1, female urologist; functional and reconstructive urology subspecialties).

#### Clinician and Patient Discomfort or Reluctance

Many participants consistently described pelvic examinations as awkward themselves and causing discomfort for patients, contributing to their reluctance to perform them. Some participants expressed the assumption that female patients would prefer not to be examined by a male clinician. Others acknowledged the potential discomfort involved but emphasized the clinical value of the examination as outweighing that discomfort. “I think female patients are more comfortable having a vaginal examination by a female doctor. I think that’s a given. The difficulty or the challenge for a male urologist…is how you negotiate that examination. I was always highly cognizant of the fact that this is an…awkward sort of situation.” (Participant ID 7, male urologist; general).

#### Failure to Recognize Pathology

Some participants tended to regard pelvic examinations as equally important as DREs, viewing the omission of such assessments and the potential to miss relevant pathology as both inexcusable and indefensible. In contrast, others questioned the clinical value of performing pelvic examinations in outpatient settings, describing pelvic pathology as benign and often favoring alternative investigations such as flexible cystoscopy or ultrasound as sufficient substitutes. “I think your medical risk is greater by not examining a patient and missing important physical pathology.” (Participant ID 7, male urologist; general).

## Discussion

Although pelvic examinations are critical to the assessment of a patient with LUTS and should be undertaken routinely as one of the first steps of their evaluation, the results from our survey of 74 urologists and trainees demonstrated that 91.3% of male clinicians and 14.3% of female clinicians do not routinely perform pelvic examinations for female patients with LUTS at their initial appointment. Qualitative data from our semistructured interviews identified themes centered on fear and barriers to these examinations.

These findings align with previous research indicating that urologists may underoffer pelvic assessments in the evaluation of patients with LUTS,^6^ with time constraints and chaperone issues identified as barriers.^13^ Our study suggested that many urologists believe that pelvic examinations for symptomatic patients have limited utility. While screening pelvic exams are not recommended for asymptomatic females,^14^ both the American Urological Association and European Urological Association guidelines strongly endorse physical examination^3,15^ in the initial office evaluation of patients presenting with LUTS. Ensuring up-to-date education of urologists and trainees is key, as well as providing them with the skills, safety, and confidence necessary to examine patients. The updated USANZ training curriculum has ensured greater emphasis on female urology,^16^ which aligns with the increasingly popular subspecialization of female and functional urology and a correspondingly increased proportion of female trainees.^17^

Many participants in our study expressed fear of causing patient discomfort during examination and anticipated that female patients would prefer a female clinician. However, for care to remain patient-centered, assumptions concerning patient preferences need to be reviewed. It has been shown that patient discomfort during pelvic examination is independent of the clinician’s gender,^18^ with bedside manner the major influence on patient experience.^7^ Clinicians should not be deterred from performing pelvic examinations in the mistaken belief that females will object. Many participants also expressed fear of medicolegal reprimand from patients for performing a pelvic examination, concerned about being accused of inappropriate behavior. As pelvic examinations are recommended by guidelines to be performed at the initial evaluation, failure to do so could expose clinicians to medicolegal risk, which aligns with some of our study participants’ concerns about the risk of missing relevant pathology. Many participants thought it logistically easier to perform a pelvic examination at the time of a flexible cystoscopy examination. However, many causes of LUTS are identifiable through pelvic examination alone. The diagnosis and management of pelvic floor prolapse, stress urinary incontinence, overactive bladder, and urethral atrophy do not require a flexible cystoscopy in its initial evaluation.^4,15^ The omission of a pelvic examination may delay recognition and treatment and result in unnecessary investigations and procedures.

In our study, a lack of access to chaperones was suggested as a barrier to conducting pelvic examinations. Chaperones may assist in providing safety for the patient and clinician by mitigating feelings of discomfort and allegations of inappropriate behavior.^19^ We found that 58.7% of male urologists and 3.6% of female urologists routinely used a chaperone when performing a pelvic examination for patients of the opposite sex, compared with 72.5% of British Association of Urological Surgeons (BAUS) urologists.^20^ However, many female patients prefer not to have a chaperone,^21^ with the majority wanting to be involved in the decision to have a chaperone.^22^ Other studies also show that more than half of patients prefer a family member or friend as a chaperone.^23^ In the survey of BAUS urologists, only 28.1% routinely asked a patient if they would prefer the presence of a chaperone.^20^ There are many guidelines offering protocols and approaches to pelvic examinations and the use of chaperones.^24,25^ The Royal Australian College of General Practitioners recommends that clinicians consider whether to involve a chaperone in a consultation on a case-by-case basis. The Royal Australasian and New Zealand College of Obstetricians and Gynaecologists suggests that clinicians ensure that the patient is fully informed and that verbal consent for the examination is obtained.^25^

### Limitations

The limitations of this study include the relatively low survey response rate; however, the study was adequately powered based on calculations conducted prior to the study. This modest response rate raises the possibility of nonresponse bias, whereby individuals with particularly strong views or direct experience of the topic may have been more likely to participate. As such, the findings should be interpreted as reflecting respondents’ perspectives rather than the entire eligible population. Nevertheless, in the context of qualitative research, the data were rich and remain valuable for generating insight, identifying themes, and informing further inquiry. The contextual factors considered are specific to the Australian and New Zealand urological surgical environment, and as such, results may not be applicable to other specialties or regions. Further investigation is needed to determine how training and continuing education programs can promote pelvic examination and overcome barriers so that female patients can receive the highest standard of care.

## Conclusions

This qualitative, mixed methods study found that male clinicians are less likely than female clinicians to perform a pelvic examination for patients in the investigation of LUTS. Barriers to pelvic examination need to be addressed to enable female patients to receive optimal care. Focused training and increased expectations may help prevent unnecessary procedures and improve patient outcomes.

## Notes

Supplement 1. **eAppendix 1.** Survey Questions **eAppendix 2.** Semi-Structured Interview and Questions

Supplement 2.
Data Sharing Statement
