# Supplementary material for: Pelvic and Digital Rectal Examinations to Evaluate Lower Urinary Tract Symptoms
Source: JAMA Netw Open. 2026 Apr 27;9(4):e269267. doi: 10.1001/jamanetworkopen.2026.9267 (PMC13122398; doi:10.1001/jamanetworkopen.2026.9267)
Supplement: Supplement 1. — eAppendix 1. Survey Questions eAppendix 2. Semi-Structured Interview and Questions [file jamanetwopen-e269267-s001.pdf]

Supplementary Online Content

McLeod K, Youssef M, Rogers M, Grills R. Pelvic and digital rectal examinations to evaluate lower urinary tract symptoms. *JAMA Netw Open*. 2026;9(4):e269267.
doi:10.1001/jamanetworkopen.2026.9267

eAppendix 1. Survey Questions
eAppendix 2. Semi-Structured Interview and Questions

This supplementary material has been provided by the authors to give readers additional information about their work.

© 2026 McLeod K et al. *JAMA Network Open*.

eAppendix 1. Survey Questions

1. What is your level of training?
    a. SET1
    b. SET2
    c. SET3
    d. SET4
    e. SET5
    f. Fellow, after obtaining FRACS (Fellow of the Australasian College of Surgeons) qualification)
    g. Consultant

2. If answered *consultant* to question 1, please include your area(s) of specialty (e.g. robotic, oncology, functional, reconstructive, andrology, etc.)

3. Do you identify as:
    a. Male
    b. Female
    c. Other

4. In your practice over the last 12 months, have you routinely (more than 75% of the time) performed a digital rectal exam in outpatient clinic/your rooms on male patients who present with lower urinary tract symptoms (LUTS)?
    a. Yes
    b. No – please provide your reasons why

5. In your practice over the last 12 months, have you routinely (more than 75% of the time) performed a pelvic floor (vaginal) exam in outpatient clinic/your rooms on female patients who present with lower urinary tract symptoms (LUTS)??
    a. Yes
    b. No – please provide your reasons why

© 2026 McLeod K et al. *JAMA Network Open*.

6. In your practice over the last 12 months, have you used a chaperone when performing a DRE?
    a. Yes, with all/most patients (more than 75%)
    b. Only on request of the patient
    c. Only sometimes – case by case
    d. No/rarely

7. In your practice over the last 12 months, have you used a chaperone during vaginal examinations?
    a. Yes, with all/most patients (more than 75%)
    b. Only on request of the patient
    c. Only sometimes – case by case
    d. No/rarely

8. Would you be happy to participate in a short semi-structured interview over zoom to discuss your answers at a time suitable to you (10-15min)?
    a. Yes
    b. No

9. If yes, please outline your preferred contact method
    a. Phone – provide number
    b. Email – provide email

© 2026 McLeod K et al. *JAMA Network Open*.

eAppendix 2. Semi-Structured Interview and Questions

Preamble

At Barwon Health, we are looking to identify the attitudes, perceptions and confidence levels of urologists and urology trainees in Victoria towards the female pelvic examination in outpatient clinics/private rooms, particularly whether there is a difference between male and female practitioners.

We have hypothesised that male trainees and urologists are less likely to routinely perform female pelvic examinations in outpatient clinics/private rooms than female urologists and trainees, and look to explore any themes that may contribute to this.

Please advise if you wish to refrain from answering any questions as we proceed.

Interview questions:

1. Introductions
2. Review of survey questions
    a. What is your level of training?
    b. If answered consultant, how many years have you been practicing?
3. What is/are your area(s) of interest?
4. How would you describe the role of a DRE in your practice?
5. How often do you perform a DRE on a male patient that presents with LUTS in the outpatient setting? Why?
6. How often would you offer a chaperone at the time of DRE?
7. What factors would prevent you from performing a DRE?
8. How would you describe the role of a pelvic (vaginal) examination is in your practice?
9. How often do you perform a pelvic (vaginal) examination on a female patient that presents with LUTS in the outpatient setting?
10. How often would you offer a chaperone at the time of a pelvic (vaginal) examination?
11. What factors would prevent you from performing a pelvic (vaginal) examination?
12. Do you find you approach invasive examinations differently with male patients compared to female patients?

© 2026 McLeod K et al. *JAMA Network Open*.

13. Do you feel your gender plays a part in how you approach male versus female patients?
14. Have you ever referred a patient to a colleague or referred a patient to have a procedure + examination (e.g. flexible cystoscopy + vaginal exam) instead of examining the patient in outpatient clinic/your rooms? (not including from telehealth appointment) Were these predominantly male or female patients? Why?
15. Do you feel male and female LUTS are adequately and equally investigated?
16. Do you have any additional comments?

© 2026 McLeod K et al. *JAMA Network Open*.
