# Supplementary material for: Pelvic and Digital Rectal Examinations to Evaluate Lower Urinary Tract Symptoms
Source: JAMA Netw Open. 2026 Apr 27;9(4):e269267. doi: 10.1001/jamanetworkopen.2026.9267 (PMC13122398; doi:10.1001/jamanetworkopen.2026.9267)
Supplement: Supplement 2. — Data Sharing Statement [file jamanetwopen-e269267-s002.pdf]

Data Sharing Statement

McLeod. Pelvic and Digital Rectal Examinations to Evaluate Lower Urinary Tract Symptoms. *JAMA Netw Open*. Published April 27, 2026. doi:10.1001/jamanetworkopen.2026.9267

Data
Data available: No

Additional Information
Explanation for why data not available: Data was considered sensitive, and participants were guaranteed confidentiality.
